# Multi-Sensor Context-Aware Based Chatbot Model: An Application of Humanoid Companion Robot

**DOI:** 10.3390/s21155132

**Published:** 2021-07-29

**Authors:** Ping-Huan Kuo, Ssu-Ting Lin, Jun Hu, Chiou-Jye Huang

**Affiliations:** 1 Department of Mechanical Engineering, National Chung Cheng University, Chiayi 62102, Taiwan; phkuo@ccu.edu.tw; 2 Advanced Institute of Manufacturing with High-Tech Innovations (AIM-HI), National Chung Cheng University, Chiayi 62102, Taiwan; 3 Department of Intelligent Robotics, National Pingtung University, Pingtung 90004, Taiwan; cbc105010@gmail.com (S.-T.L.); wuorsut@gmail.com (J.H.); 4 Department of Data Science and Big Data Analytics, Providence University, Taichung 43301, Taiwan

**Keywords:** multi-sensor fusion, natural language, processing, context-aware computing, chatbot, companion robot

## Abstract

In aspect of the natural language processing field, previous studies have generally analyzed sound signals and provided related responses. However, in various conversation scenarios, image information is still vital. Without the image information, misunderstanding may occur, and lead to wrong responses. In order to address this problem, this study proposes a recurrent neural network (RNNs) based multi-sensor context-aware chatbot technology. The proposed chatbot model incorporates image information with sound signals and gives appropriate responses to the user. In order to improve the performance of the proposed model, the long short-term memory (LSTM) structure is replaced by gated recurrent unit (GRU). Moreover, a VGG16 model is also chosen for a feature extractor for the image information. The experimental results demonstrate that the integrative technology of sound and image information, which are obtained by the image sensor and sound sensor in a companion robot, is helpful for the chatbot model proposed in this study. The feasibility of the proposed technology was also confirmed in the experiment.

## 1. Introduction

Many studies have proposed robots that can chat with people. Opportunities to use chatbots will continue to increase. In addition to accompanying older adults living alone, chatbots are also applied to medical treatment. For example, individuals may be afraid of public spaces due to mental illnesses. Under these circumstances, chatbots can be used to provide them with companion and health care. Numerous studies of chatbots have been reported. Traditional chatbots only use the before and after sentences for training. However, such training results in low diversity, and accuracy is also low. This traditional system also renders chatbots unable to flexibly answer questions. For example, when being asked “What do you see?” traditional chatbots can only answer “I don’t know” due to the lack of image information. The aforementioned problem is only one example; numerous others are similar.

Numerous studies regarding chatbots have been published. Ref. [1] proposed and applied a hybrid chatbot to communication software. This technology combines a retrieval-based model and a QANet model and can answer user questions through an E-Learning system. In additional to supporting English, this technology can also converse with chatbots in Chinese and has performed well in experiments. Ref. [2] also applied chatbots in digital learning. In this study, the role of the proposed chatbot is to resolve Visual, Auditory, Read, Write, and Kinesthetic (VARK) information throughout the process of responding to the learner. Furthermore, the proposed chatbot also records the learner’s beta brainwaves and uses machine learning algorithms to classify learning orientations. Ref. [3] explored many studies on chatbots used in Facebook Messenger, mainly discussing how teachers use chatbots to supplement their teaching. The article also stated that the use of chatbots to assist in teaching is not yet mature and, therefore, chatbots as a field of research offer considerable room for development. Ref. [4] proposed a neural network-based chatbot that integrates personal data for analysis and estimation and uses the useful data to return appropriate responses. The main architecture for this technology is LSTM [5], and experiment results have substantiated the feasibility of this technology. In Ref. [6], bidirectional recurrent neural network (BiRNN) technology was used as the basis for a chatbot, and a GRU [7] was used as the core of the technology. The system architecture was exceedingly large and required substantial computing resources, but was verified favorably in experiments. Ref. [8] proposed a chatbot called Xatkit and stated that although it requires improvement, it currently can be applied in some real-world scenarios, and could be applied in many venues in the future.

Chatbots have diverse applications. To address the massive demand for manpower in customer service, some companies have proposed automated customer service chatbots. However, automated customer service chatbots sometimes misunderstand sentences. Ref. [9] presented research into automated customer service chatbots and stated that although chatbot technology is not yet fully mature, demand for this technology is notably high. Moreover, cognitive behavioral therapy (CBT) is a common method of therapy for patients with panic disorder. Ref. [10] explored the feasibility of using chatbots on mobile devices for CBT. The study found that using chatbots for CBT was both feasible and effective, pioneering the application of chatbots in medicine.

Deep learning as a field of research has burgeoned in the early 21st century. Its technological developments are diverse and have been applied to many research topics [11,12,13,14,15]. The present paper features deep learning techniques to resolve questions that chatbots cannot answer due to environmental contexts. The first step toward this type of practical learning is to enable bots to understand the present environment through visual images and to describe the environment through text. The most direct method is to design bots that use image caption technologies to understand their environments. The original purpose of image captions was, and is, to automatically generate annotations for an image. For example, if an image is entered as input, the image caption model can write a sentence about the image based on the image status. Therefore, in this article, the described method enables the bot to view its present environment clearly and to know about any object in its environment. Conventional chatbot models typically are only able to perform model training based on context; this type of model training is unable to consider image data. Even if the model can understand its present environment, it cannot incorporate the understood image data into dialogue responses. In this study, the proposed method can deepen the chatbot model’s considerations of the results of its previous conversations, and together with the current dialogue, can train the chatbot. A statement generated by image caption technology can also be an input for the chatbot and can be incorporated into the next dialogue. This method enables the chatbot to answer questions more accurately; the highly diverse responses vary based on the present environment.

The four major contributions of this work are: (1) integrating image and sound data to achieve further precision and practicality in chatbot and human communications; (2) adjusting and optimizing a framework based on a commonly used LSTM model to train the image caption and chatbot model efficiently, thus improving scenario recognition abilities; (3) comparing the original LSTM-based model with the adjusted, GRU-based version and verifying that the training efficiency was improved by the adjustments; and (4) testing the trained model on a humanoid robot in a series of experiments to verify that the other model proposed in this paper and the applications of both models are effective and feasible.

The remaining sections of this paper are as follows. Section 2 details the operational concepts and process of this paper. Section 3 introduces the models and algorithmic framework being proposed. Section 4 presents and compares the experiment results. The discussions are addressed in Section 5. Section 6 is the conclusion.

## 2. System Architecture

In the past, chatting with bots was typically only possible through direct voice or text conversations using conventional chatbot models. A typical bot was unable to consider its surrounding environment through its vison when responding. The present research relies on chat training with images that the bot can see. To allow the chatbot models to integrate image data into input sets, this paper proposes an improved model that integrates image captions, natural language processing, and similar technologies; this model enables the bot to chat and respond according to the environment it sees. The feasibility and practicality of the model were substantiated by the experiment results. In the future, this method can be applied to chatbots and home care, as well as to increase the precision of typical chatbot’s verbal expressions and to enable typical chatbots more aware of reality when conversing with humans.

**Figure 1:**
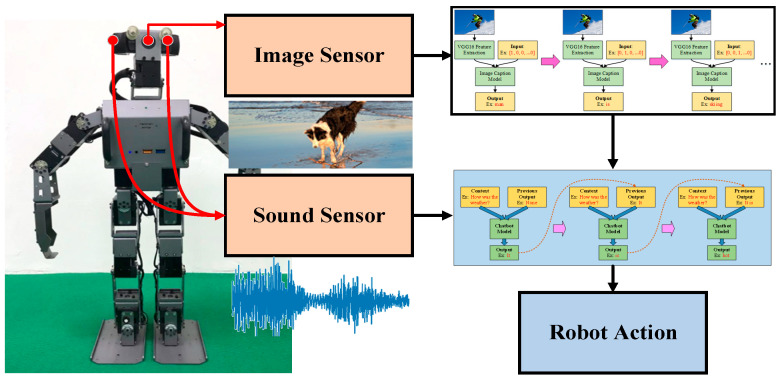
An overview of the proposed framework.

**Figure 2:**
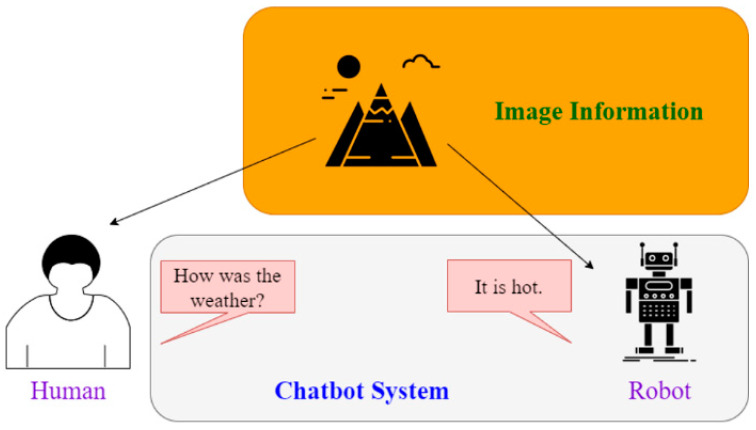
The concept of the proposed framework.

The overview of the proposed framework is illustrated in Figure 1. Prior to undergoing any training, the chatbot can be said to be like a baby—incapable of anything. But after going through correct learning, the chatbot slowly becomes more human-like and gains the ability to communicate with people. The training process resembles the process by which adults teach children to recognize objects, typically holding a picture and telling the child, “This is a dog”. Although the ability to express oneself in words may not be entirely present during the initial training, the rate of failure can be reduced through continuous training. Therefore, we can use this process to enable robots to develop cognitive abilities regarding their surroundings. This process is the same as teaching children to speak, that is, developing their ability to speak through continuous conversations and correcting their mistakes. Teaching robots is the same—although the bot may answer incorrectly in the beginning, through continuous learning, they can learn to have normal conversations with people. The concept map of this paper is presented in Figure 2; the chatbot can perform analyses based on current image data. When the user asks questions, not only is the chatbot able to make considerations based on the environmental data it currently detects, the bot is also able to generate a corresponding response based on the conversation context. The benefit of this method is in the integration of multiple pieces of data, allowing the chatbot to better make responses focusing on relevant questions. In this manner, the bot’s responses become more precise and more in line with reality.

**Figure 3:**
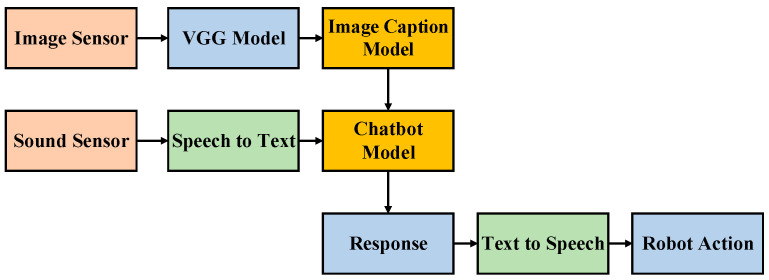
The architecture of the system.

This paper can be split into two major areas. The first is picture scenario analysis, and the second is natural language processing. The system framework is presented in Figure 3. When the entire system is operating, we consider two inputs: image data and voice inputs. For the image inputs, first, we perform feature extraction through the VGG model. This reduces the dimensionality of the image data, avoiding training problems resulting from overly large input dimensions. Following the feature extraction, we then use the image caption model to generate a paragraph of text that matches the image data. For the voice inputs, we must first convert the sound signals into text messages through voice-to-text conversion technologies. Then the chatbot model must generate appropriate responses based on text messages converted from the user’s voice and text information about the pictures. In this context, the output data type is text. Therefore, for the user to be able to hear the chatbot’s response, we use text-to-voice conversion technologies to convert the text message into sounds, either to send to the user or to enable the bot to perform a corresponding action. In this manner, the whole system framework not only considers signals from sound, it also incorporates considerations of images. Therefore, the proposed method improves chatbot affinity by enabling chatbots to generate responses to be more in line with reality and to interact better with humans.

**Figure 4:**
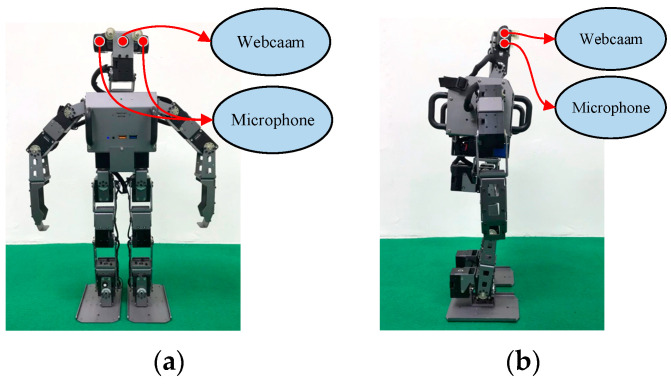
The architecture of the humanoid robot: (**a**) the front view; and (**b**) the side view.

To validate the method proposed in this paper, we applied the trained models with a humanoid robot, then verified the efficacy of the method through a series of experiments. Humanoid robots are the closest robot type to humans and are therefore suitable for chatting and companionship purposes; therefore, a humanoid robot was chosen for the experiments in this paper. The humanoid robot used in this paper is presented in Figure 4; the humanoid robot is 51 cm tall and weighs approximately 3.5 kg. It has 20 degrees of freedom, and has an inbuilt Logitech C920 HD Pro Webcam and microphone. The robot is powered by a XM430 motor and installed with an Intel Core i3 processor and OpenCR controller. Its Inertial Measurement Unit involves a three-axis gyroscope, a three-axis acceleration sensor, and a three-axis magnetometer. The operating system is Ubuntu.

## 3. The Proposed Model

This section introduces the framework of the proposed model in detail. Because most of the data processed in this work was time-series data, RNNs constituted an appropriate framework. The most well-known RNN framework is the commonly used LSTM. The literature currently features many LSTM variations, such as the GRU framework. However, the GRU framework is simpler than the LSTM framework, but in actual operations the GRU can achieve results that are equal to (if not better than) the results of LSTM. Also, because the GRU framework is simpler, under normal conditions GRU has better training efficiency than LSTM. To compare the two RNN models, we must modify and enhance the original LSTM RNN in the hopes of achieving better convergence efficiency than the original neural network.

**Figure 5:**
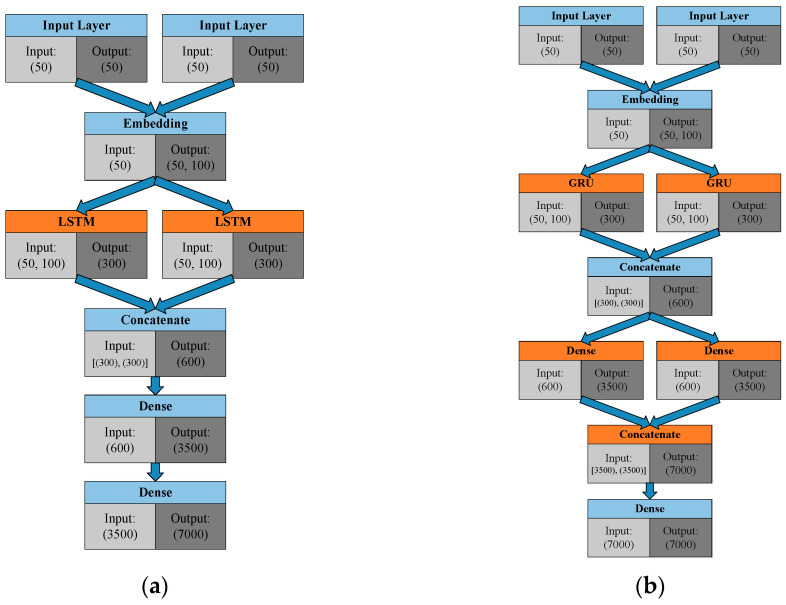
Architecture of the chatbot: (**a**) LSTM version; and (**b**) Enhanced GRU version.

Many studies have performed in-depth explorations of chatbots, and this paper takes the framework from reference [16] as the basis for improvements. In the original version, an adversarial learning method for generative conversational agents (GCA) is applied for the chatbot model. Such a learning process originally also belonged to one of the sequence-to-sequence training regimes. However, such an approach can also transfer to an end-to-end training process by applying the specific structure. The chatbot model can generate an appropriate sentence by considering the dialogue history. The main core of GCA is LSTM, and the model also considers the current answer and the previous conversation history. The concept for this paper also was implemented with favorable results in the Keras framework [17], as presented in [18]. The Keras framework is a deep learning library which includes many Application Programming Interfaces (APIs) for building the corresponding models. Keras is also one of the most used deep learning frameworks. In [18], Keras is used for the implementation of the chatbot model. Based on the previous work in [18], this study improves the model structure and gives better performance than the original version. The detailed framework of the chatbot is exhibited in Figure 5; Figure 5a presents the original LSTM version [18]; Figure 5b displays the GRU version that was optimized in this study. In addition to the LSTM-to-GRU transformation, the differences between the two versions include the addition of two dense (fully connected) layers to the new version. The parameters are listed in Table 1. We also added a dropout to prevent overfitting and used Adam for the optimizer and categorical cross entropy for the generated loss function. A more detailed comparison of the two versions is laid out in the next section.

The chatbot model proposed in this study was based mainly on the concept of a generative conversational agent (GCA) [16]. Records of a conversation with a chatbot can be stored using the *x* vector and one-hot encoding. Accordingly, the following equations are defined:

(1) $$X=\left[{\bar{x}}_{1},{\bar{x}}_{2},{\bar{x}}_{3}\ldots {\bar{x}}_{S_{s}}\right]$$

(2) $$Y=\left[{\bar{y}}_{1},{\bar{y}}_{2},{\bar{y}}_{3}\ldots {\bar{y}}_{S_{s}}\right]$$

(3) $$E_{c}=W_{e}X$$

(4) $$E_{a}=W_{e}Y$$

where *W_e_* represents the embedding layer parameter, *E_c_* represents the corresponding conversation record, and *E_a_* represents the unfinished response. An RNN is then used to extract the embedding vectors of the conversation record and response, as illustrated in the following equation:

(5) $$e_{c}=\Gamma_{c}\left(E_{c};W_{c}\right)$$

(6) $$e_{a}=\Gamma_{a}\left(E_{a};W_{a}\right)$$

(7) $$e=\left[e_{c}\;e_{a}\right]$$

(8) $$y_{h}=\sigma \left(W_{1}e+b_{1}\right)$$

(9) $$P=\varphi \left(W_{2}y_{h}+b_{2}\right)$$

where *W*_1_ and *W*_2_ are synaptic weights, *b*_1_ and *b*_2_ are biases, *σ* is the Rectified Linear Unit (ReLU), and φ is the Softmax activation function. Finally, the index with the largest probability value is identified from *P* before the next iteration is started. Accordingly, the most suitable response can be output.

**Figure 6:**
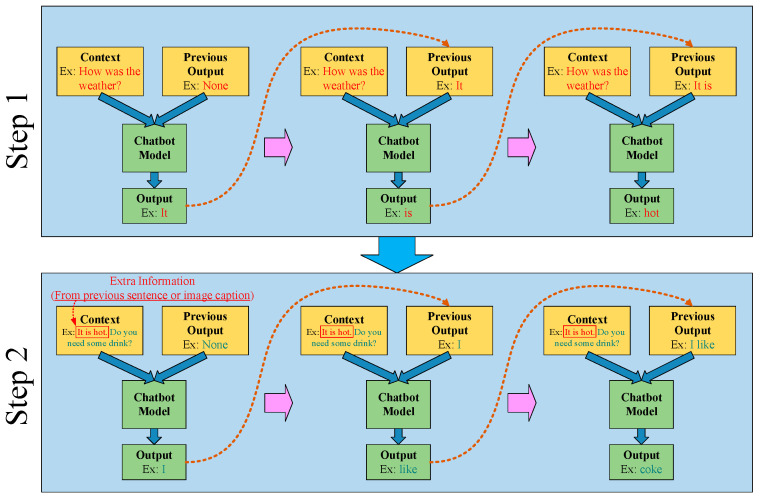
The workflow of the designed chatbot model.

The operational method of the chatbot used in this paper is displayed in Figure 6. The figure displays the two input sources of the chatbot. The left-hand input source is the previous sentence spoken by the other party, and the right-hand input source is a collection of previous output results. Because of the unique advancements of the proposed training, the chatbot model can notably understand and analyze the contextual relationships of the sentences. Finally, we export the generated dialogue word-by-word in sequence, then assemble the words into a complete sentence. When the chatbot expresses the output results for the user to hear, the user may respond a second time based on the answer just received. Next, in addition to the second response from the user, the chatbot’s own previous response is added to the chatbot’s left-hand input. This additional input is exceedingly helpful to the chatbot’s ability to understand the contextual meaning. Furthermore, this additional input can be substituted for the output text produced by the image data. This allows the chatbot to become familiar with the present image data, enabling the chatbot to assess the image content and generate the best response. Through repeated training, the chatbot model can fully grasp the relationship of the conversation with the user; in this manner, the chatbot can become more fluent in conversations with people, narrowing the gap between chatbots’ and humans’ communication skills.

**Figure 7:**
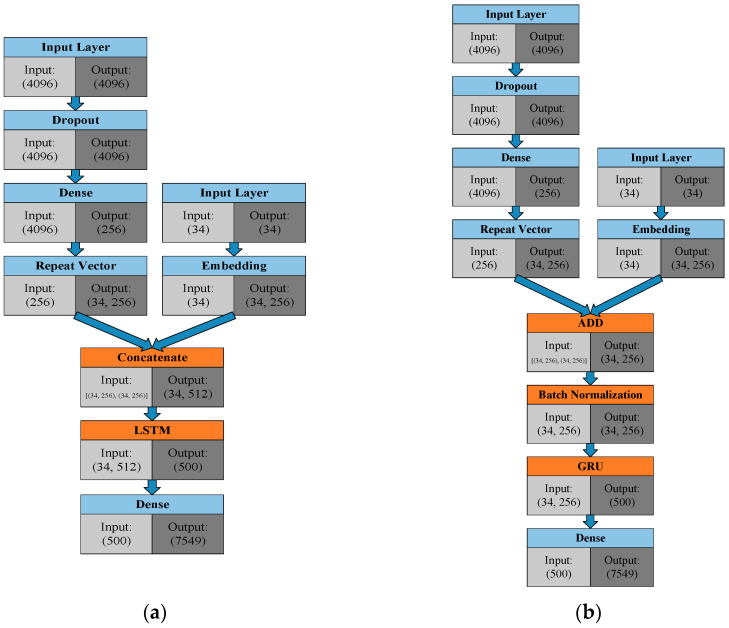
Architecture of the image caption: (**a**) LSTM version; and (**b**) Enhanced GRU version.

Image caption technology has been a topic of in-depth exploration in many papers, and this paper builds on the framework in references [19,20]. The concept discussed in this paper was also implemented with favorable results in the Keras framework [17], as demonstrated in [21]. A detailed framework of our image caption model is presented in Figure 7, with Figure 7a as the original LSTM version [21] and Figure 7b as the optimized GRU version used in this paper. The original LSTM framework was exchanged for a GRU framework, to reduce the number of training parameters; the concatenation layer that had originally been in the middle was implemented through a tensor adding method. The improved approach, in addition to being able to consider input data from both sides, can also eliminate half of the parameters for this layer and improve training efficiency. Furthermore, batch normalization (BN) techniques were added to the model; BN can adaptively adjust the value range of the input data, reducing situations in which the activation function cannot be calculated as a result of the value of the previous layer being too great or too small. The parameters of the image caption model discussed in this paper can be found in Table 2. We also added dropout to prevent overfitting; we chose Adam for the optimizer and categorical cross entropy for the output loss function. The next section compares these two versions in greater detail.

**Figure 8:**
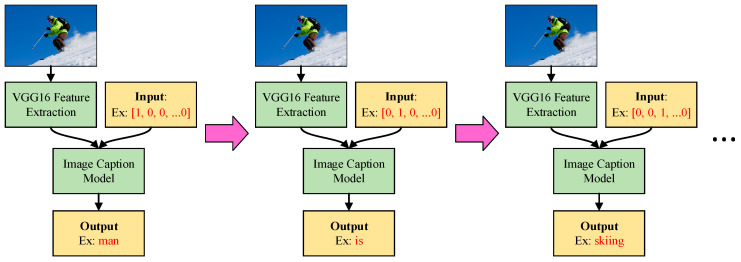
Workflow of the designed Image caption model.

Figure 8 presents the operations of the image caption model; the figure demonstrates that the image caption model can also be split into left-hand and right-hand inputs. The left-hand inputs are image-related; after an image is input into the model, we must first use VGG16 [22] to perform the task of feature extraction. The VGG16 model used in this paper is a complete model that has already been trained using a large volume of picture data. The final output layer of the original was not necessary for this work; but because this model had already been trained, the final output layer was eliminated, and the other parts were treated as excellent feature extraction tools. If we input an entire picture into the image caption model from the beginning, this may result in training issues from overly large input data. The use of the feature extraction method reduces the input dimensions and can present the picture’s data in the most streamlined and adequate manner as vectors. The image caption model’s right-hand inputs are a series of one-hot vectors that represent the current sequence of the output text. After repeated iterative processes, the image caption model describes the image in text by generating the text word-by-word to present a complete sentence.

**Figure 9:**
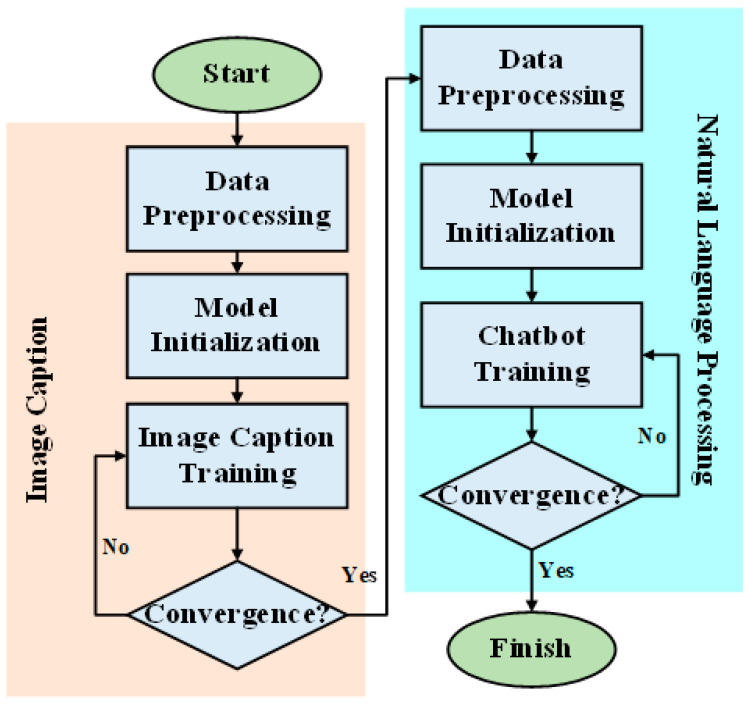
System flowchart of the training process.

The model training process discussed in this paper is presented in Figure 9. The detailed pseudocode is listed in Algorithm 1. The details are addressed in Appendix A.
**Algorithm 1: The procedure of the proposed system.**1.Data preprocessing (image caption)2.Image caption model initialization3.**for** all training epochs4.  **for** all image caption training samples5.    Training process6.  **end for**7.**end for**8.Data preprocessing (chatbot)9.Chatbot model initialization10.**for** all training epochs11.  **for** all chatbot training samples12.    Training process13.  **end for**14.**end for**

The process can be split into two major sections: the first section is the image caption model, and the second part is the natural language processing part. In the image caption model, we first perform preprocessing on the data by consolidating and coding all the image data and the labeled text data. Then, after we initialize the model, we conduct model training. In the training process, we must observe whether the convergence conditions have been satisfied; if not, then training must be continued. If the text caption model training is complete, then the process enters its next stage. In the natural language processing part, the first and most crucial task is still the data preprocessing, followed by the initialization of the chatbot model. Next, training of the chatbot model must be performed using the processed data. Similarly, if the chatbot model does not satisfy the convergence conditions, the training must continue. Conversely, if the chatbot model has satisfied the convergence conditions, the training immediately ends. When the convergence conditions are met, the entire training process is complete. However, the image caption model must be trained first because this model plays a key role in incorporating image information in the chatbot input. If the image caption model is inadequately trained, the image information input into the chatbot becomes meaningless and may result in erroneous interpretation. After the training of the image caption model is completed, it can be used to convert image information to textual information, which is then fed to the input end of the chatbot. This conversion approach enables a chatbot model to be constructed without substantial modification. This also allows the model to interpret image information, which is a primary focus of this study. The problem of overfitting was considered during the training process, which was terminated immediately when overfitting occurred.

## 4. The Experimental Results

The first part of this section is a detailed explanation of the training data. Next, we present training and comparisons based on the model proposed in the previous section. Finally, we describe how the consolidated functions were integrated with the humanoid robot to test the effectiveness of the proposed model and analyze the feasibility.

### 4.1. The Training Data

**Figure 10:**
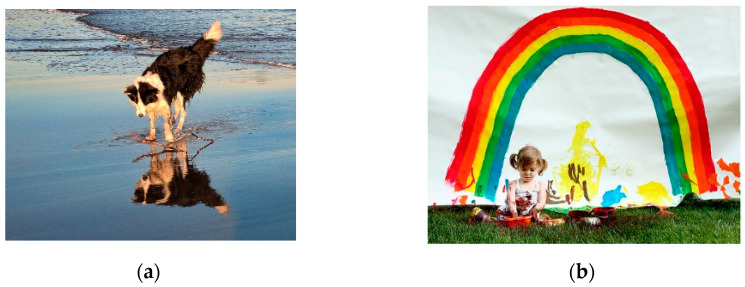
Training samples in Flickr 8 k [24]: (**a**) caption: “A dog at the beach”; and (**b**) caption: “A little girl covered in paint sits in front of a painted rainbow with her hands in a bowl”..

The databank for sentence training in this paper consisted of data sets of dialogue from online English classes [18]. Although image caption technology typically uses COCO data sets [23], which involve substantial amounts of data, as the training data, this experiment used the Flickr 8k Dataset [24], which has a smaller data volume, so that the model training results can be used with actual robots that may not have rapid computing abilities. The Flickr 8k Dataset has 8000 color images in total, and each image has five captions (see Figure 10 for the training data). From the images, we can see that each image has a chunk of text that describes the picture; these descriptions are man-made captions. Therefore, each image has various appropriate descriptions. Therefore, one of the key points of this paper is that image data must be described in text and incorporated into the chatbot’s considerations. In this manner, conversations between humans and bots can become more precise and more in line with actual scenarios. Furthermore, in addition to the data mentioned, some new images were also added to the experiment in pursuit of greater realism and more accurate representation of the experimental scenario.

### 4.2. The Experimental Results

**Figure 11:**
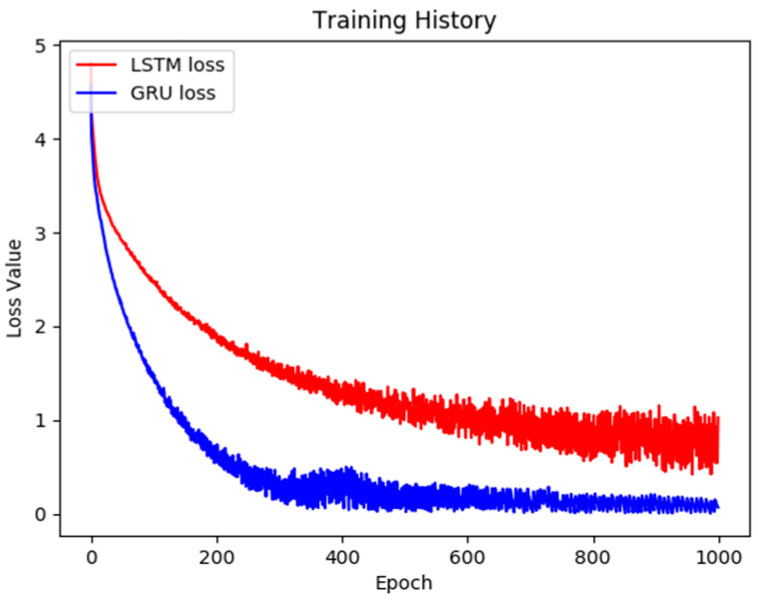
The learning curve of the chatbot model.

**Figure 12:**
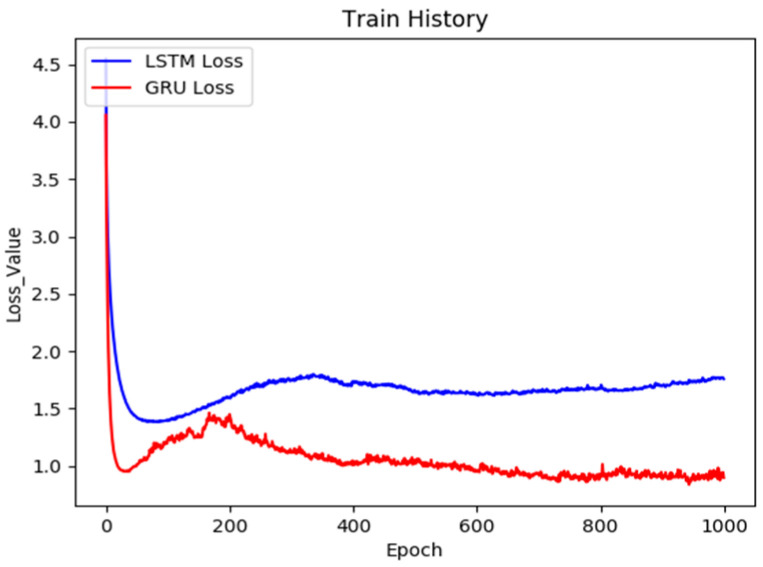
The learning curve of the image caption model.

To discuss the strengths and weakness of the proposed model and the original LSTM model in depth, we made the following comparisons. The chatbot training models are compared in Figure 11, whereas the image caption training models are compared in Figure 12. The figures demonstrate that during the chatbot model training process, the proposed method is faster and stabler and has a lower final training error rate. This also substantiates that the model proposed in this paper outperforms the original and conventional frameworks. However, during the training process of the image caption model, the performance of updated model proposed in this study was also slightly better. Although the error value sometimes increased slightly during the training process, overall it did not affect convergence. The final convergence results proved that the method proposed in this paper significantly outperformed conventional chatbot models. Conventional chatbot models, even in the later stages of training, exhibit trends of increasingly higher error values. This phenomenon once more substantiates that the stability and the validity of the method proposed in this paper are excellent.

**Figure 13:**
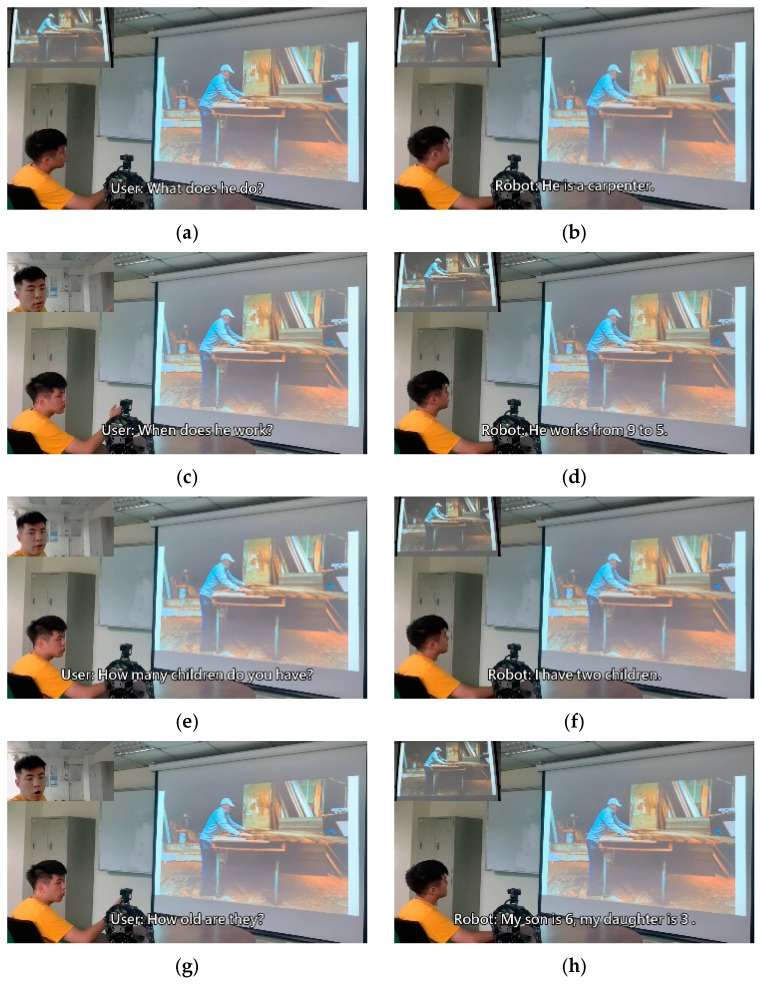
Experimental result in scenario A.

**Figure 14:**
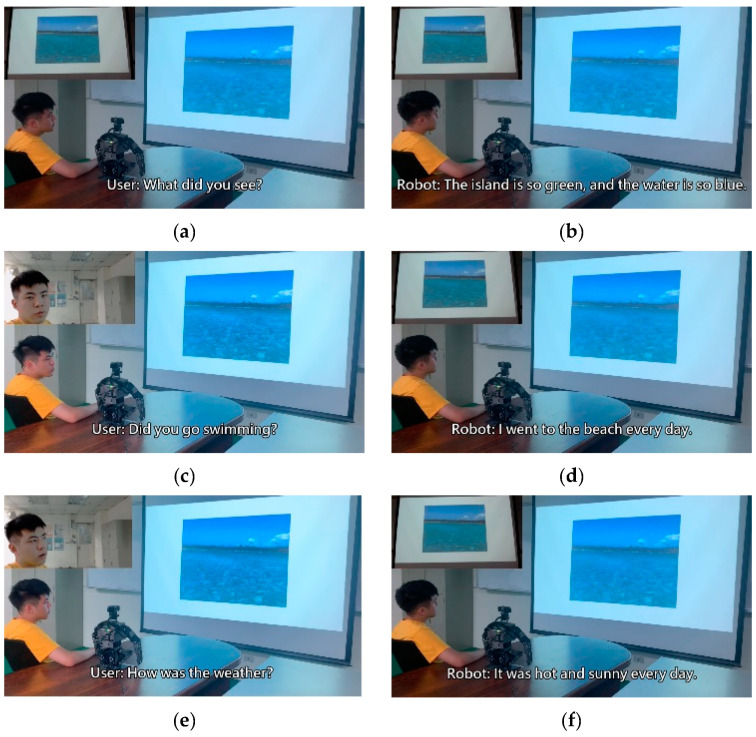
Experimental result in scenario B.

**Figure 15:**
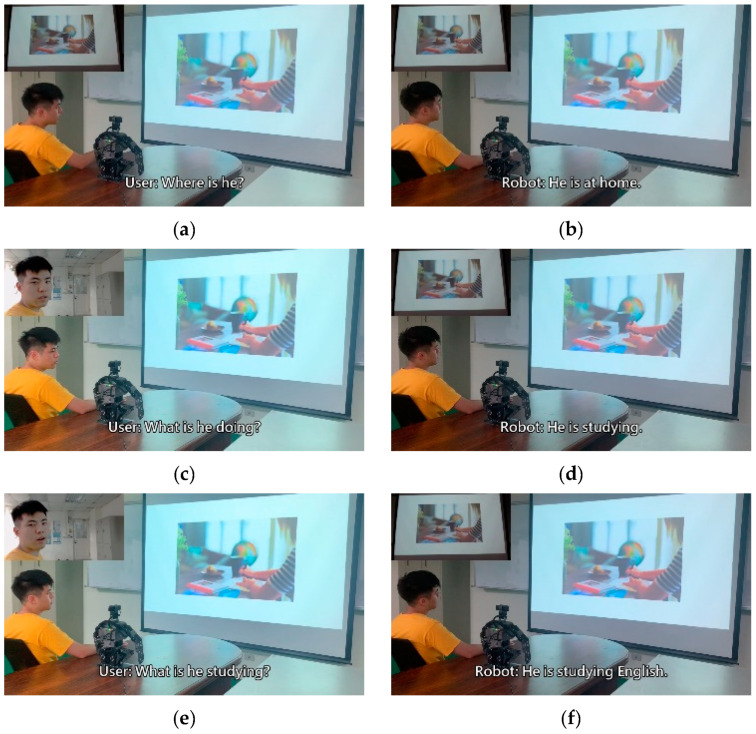
Experimental result in scenario C.

**Figure 16:**
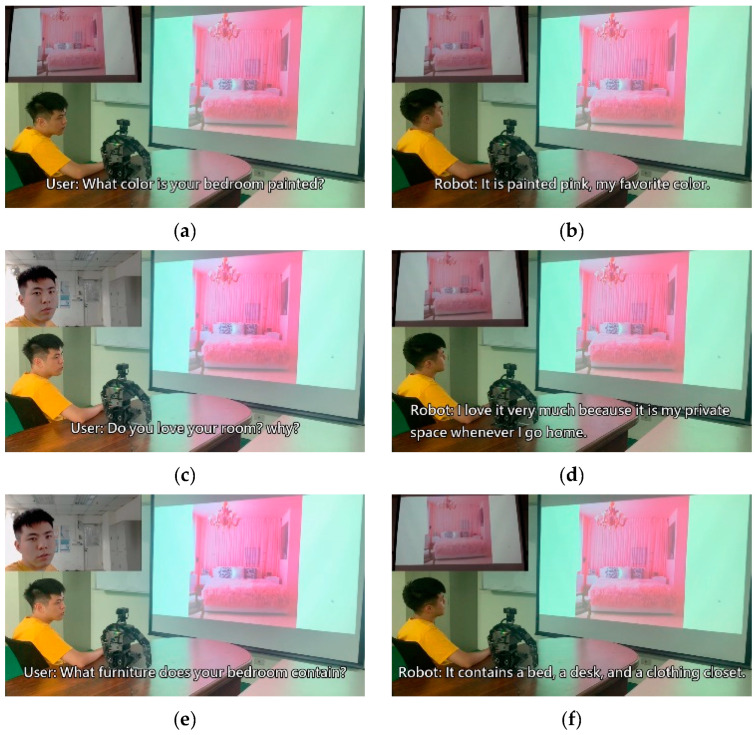
Experimental result in scenario D.

To verify the practicality of the method proposed in this study, we incorporated a humanoid robot system into the following experiment and integrated the trained model into the humanoid robot. The experiment was split into a total of four scenarios; each scenario was matched with a picture to represent the present situation. The bot, based on the situation and user dialogue in the moment, made a corresponding response. Therefore, the bot’s inputs were not limited to the user’s voice data, but also included image data. The experimental results are depicted in Figure 13, Figure 14, Figure 15 and Figure 16.

The experiment combined image caption with natural language processing technology. The robot was expected to give corresponding responses through related image and sound information. In the experimental procedure, the robot used a microphone to capture sound and converted sound into text through the speech-to-text (STT) technique. Then, the robot converted the obtained image information into text by using a deep artificial neural network. Sound and image information was converted into textual information for storage then inputted to the natural language processing model. The robot was able to give reasonable responses according to the information converted from image and sound. After the robot computed suitable sentences, it used the text-to-speech (TTS) technique to convert textual information into sound information. Thus, the text was read to allow the user to understand the robot’s response.

In some instances, the robot’s responses were not perfect. These imperfect responses may have arisen because the databank of image data and dialogue data was insufficient and, therefore, the robot was unable to fully consider all circumstances relevant to the required response. In the future, the model may process dialogue records that combine images; the model may then integrate larger and more comprehensive chatbot training databanks to increase the fluency of communications between humans and robots.

## 5. Discussion

In this experiment, the algorithm developed was integrated with a humanoid robot to facilitate conversation with humans. The robot used its built-in microphone to capture users’ voice signals in addition to using the image sensor installed on its head to capture image information in the surrounding environment. Because the robot used in the experiment features multiple degrees of freedom, it is capable of aligning its sight with that of the user to converse. Moreover, it can turn its head to observe the surrounding environment while conversing. Accordingly, the robot uses the image–text conversion model to incorporate relevant textual information into the chatbot input. The content of the conversation revealed that the response provided by the robot was associated with the image information it received. This indicated that the robot does not merely consider the relationships between voice signals when providing a response. This experiment verified that the proposed chatbot model is highly applicable to humanoid robots with companionship functions. The experimental video can be accessed in [25].

Two neural networks were used in this study, namely the chatbot model and image caption model. The main function of the image caption model is to convert implicit information in images and express it as text. The chatbot model interprets user conversations and previous responses to provide the user with the most suitable reply. The input of the chatbot model considers not only previous responses but also the text information output from the image caption model (e.g., the left input of the chatbot displayed in Figure 6). Therefore, the input end of the chatbot model retains both the information regarding the previous response and the text information output from the image caption model. This is equivalent to incorporating image information for model computation and interpretation. Accordingly, this not only resolves the problem concerning the lack of image and voice data when generating a response but also provides a convenient approach to integrate image and voice data for subsequent processing by the chatbot model. The feasibility of this approach was verified in the experiment.

In this study, dialogues from online English classes were used as the main conversation training data, whereas the Flickr 8k Dataset was used to train the image caption model. Because the conversation training data were in the form of textual information, image information first had to be converted to textual information before it could be input into the chatbot model. This conversion process was achieved using the image caption model, which was trained using the Flickr 8k Dataset before it became capable of image–text conversion. The converted textual information was then input into the chatbot model, allowing the model to consider such information when performing interpretation. This is equivalent to indirectly inputting image information into the chatbot model. This method converts all image information to textual information, facilitating information integration without the need for substantial modification to the model framework or parameter configuration. In addition, few training datasets currently exist for models that combine conversational text data and image data. Therefore, the proposed method was developed for effective training of the chatbot model through an existing conversational training dataset and image information. In the future, more training datasets can be included to improve the quality of conversations generated by the proposed chatbot model. This will further improve the applicability of the model to companion robots.

The main contribution of this study was the proposal of a chatbot model that integrates voice and image data to provide responses. This model can be applied in physical humanoid robots, allowing them to converse with humans. The experimental results revealed that, when no notable interferences existed in voice and image signals, the robot could engage in conversations fluently in most situations. Whether the responses provided by the humanoid robot can be considered accurate or fulfill user needs depends on the subjective evaluation of individuals. Consequently, because of the presence of various human-related factors, an objective quantitative evaluation of the chatbot model’s accuracy was difficult. Nevertheless, the effectiveness of the model used in this study has been verified in other studies, such as [16], indicating that the public has mostly recognized the functionality of chatbots. The model used in this study was modified based on that in [16] and therefore exhibited more favorable performance and converged faster than did the original model. In addition, the modified model demonstrated more favorable learning efficiency. The experiment performed in this study also provided strong evidence to verify the effectiveness of the proposed model.

Advancements in medical technology have made aging societies inevitable. The home care for older adults living alone is a pressing social issue. Human beings generally live in groups, and people need to converse with others. If an elderly individual has no company at home, this has a large psychological influence. Therefore, building a chatbot model for the elders may solve the above problem. The developed technique in this study can also be one of the solutions of the above issue.

In this study, the proposed chatbot model is executed in the humanoid robot. However, this model can also be executed in any computer. The processing speed is related to the computing core level of the processor. In this study, because the user will communicate with the chatbot model in the experiments, we chose the humanoid robot for the platform. During the communication, the robot can also turn its head to look around and observe the environmental information. The humanoid robot can also execute some motions to react to the user. However, if the user has no humanoid robot for the platform, a simple computer is also feasible. Therefore, the requirements of this approach are not too high. Most people can access this chatbot model.

The validations of the proposed model are shown in the experiments. The functions of the proposed framework are completely demonstrated by applying the four scenarios. In the experiment, the robot can hear what the user says and use the SST technique to recognize the speech signal. The humanoid robot can also look around to observe the environmental information for the image input. Based on the previous sentences and the current information, the robot can give corresponding responses and reactions to the user. All the functions are intergraded in the humanoid robot and validated in the experiments.

In this paper, the output of the proposed chatbot model is a sentence for the response. It cannot be treated as a classification or normal regression problem. Therefore, defining the accuracy evaluation method for the proposed chatbot model is not applicable in these cases. For evaluating the performance of the proposed enhanced models, we use the learning curves for the comparisons. The learning curves not only indicate the convergence speed, but also show the training loss. The training loss of the proposed model can also prove that the error values are also lower than the original models. Besides, for presenting the actual performance of the trained model, performing the experiments with designed scenes may be better. As a result, our proposed model gives good results in the six experiments, as shown in the experimental video provided in this paper. Moreover, the experiments also show the feasibility and superiority of the proposed model.

There are two main cores in this proposed framework. The first one is the image caption model. The image caption model is used to extract the features and transfer these features to a one-sentence caption. Therefore, this model can help the humanoid robot to understand the content of the image. However, the image caption model should also be well trained. Hence, the Flicker 8k dataset is applied for training the image caption model. This dataset includes many pictures with the corresponding caption sentences. By using this dataset, the image caption model can be well trained for generating the corresponding sentence. The other important part of the proposed framework is the chatbot model. In this part, the generated sentence will also become one of the inputs of the chatbot model. Considering the chatting history and the generated sentence, the chatbot model can give the user the correct responses. However, the chatbot model also must be trained by the collected dataset of dialogue. By combining these two well-trained models, the whole proposed framework can talk with the users. This is also the reason why the robot can understand the meaning of the picture and give the correct response to the user.

In this paper, the humanoid robot is just one of the platforms of the proposed model. The proposed model can also be executed on just a computer. However, because the humanoid robot has many degrees of freedom, the robot can interact with the user by the motions. Furthermore, the robot can also track the human face while the user is talking (as shown in the experiments). The conversation between the robot and the human sounds relatively reasonable, because the training process of the proposed model is based on the human dialogue histories. The robot can learn the “rules” of the human conversations. Besides, the prediction results also adopt the concept of the probability for selecting the best response to the user. It is hard to say that using a humanoid robot is necessary in this example. However, we think that using a humanoid robot for this application might be a good choice.

There are many researches about companion robots for helping the elderly. In [26], a companion robot is designed for the elderly person. Such a robot can operate in assistive environments in a smart home. The experiments indicate that such a system is both useful and useable. Literature [27] discusses assistive social robots in elderly care. This article mentioned that social robots are useful in eldercare. Moreover, the robot can also increase the quality of life. Literature [28] proposes a smart companion robot for elderly people. This robot applies the cloud computing technologies and fuzzy logic for the proposed robot. The experiments shows that the proposed robot system is feasible for human users. Literature [29] proposes a companion robot interaction system for supporting elderly people. Such a robot can also recognize the emotions of the users by using image processing techniques. Literature [30] proposes a home robot for elderly people with cognitive impairment. Such a robot can interact with an 89-year-old lady, and also give good results in the experiments. Literature [31] discusses the influence of social presence on acceptance of a companion robot by older people. The robot named “iCat” is applied for the experiments. The robot iCat with touch screen can help the elderly by the interactions, and the results are also validated in the experiments. Literature [32] proposes an assistive robot for older adults. Such a robot applies speech recognition techniques in the experiments. The user can also play games with the proposed robot. Based on the above researches, companion robots are very important and helpful for the elderly. In this paper, our humanoid can also be one of the companion robots to help elderly people.

However, in this paper, the humanoid robot is just a platform of the chatbot model. The proposed approach might be used for elderly people in the future. The proposed model can be executed in just one computer. It is unnecessary to perform the model in only humanoid robots. However, if the chatbot model is integrated in a humanoid robot, the humanoid robot can also perform some expressive motions during the chatting process. This might increase willingness to use this system. Therefore, we believe that the user somehow benefits by performing the chatbot model in a humanoid robot.

Although there are currently many studies that allow robots to use audio and visual data simultaneously to analyze information and make appropriate responses, in this study, the audio and visual data are analyzed in advance using different models, and the results of the analysis are input into the chatbot model. In order for the chatbot model to consider the visual data, we converted the image into text so that the robot can recognize the content of the image and use it as one of the points of reference for its next reply. This approach is still rare in current research and it is also the novelty of this research.

The goal of this research is to allow humanoid robots to have the ability to converse with humans. Through the aid of visual images the robot can perceive the external environment for it to make more correct judgments. Therefore, the output of the model proposed in this study is a string of words, which is difficult to define through comparative metrics. Consequently, to measure the effectiveness of the model, we drew a convergence curve and compared it with the model architectures proposed by other researches. According to the experiment results, the convergence effect of our proposed model is better, and this is also one of the main contributions of this paper.

In this research we use general STT and TTS methods for the audio signal processing model and have not optimized or modified the methods. The main targets for model optimization in this research are the image caption model and chatbot model used in image processing. Experiment results show that the performance of the modified model in this paper is better than that of the original model.

The image used in the experiment video is one of the images used during training in the image caption model. The light and shadow changes of the image do have a very big impact on the robot’s analysis process, therefore during the experiment, we tried to keep the ambient light in a more stable state. As for how to reduce the impact of ambient light on image analysis, it is one of the goals that we urgently need to overcome in the future.

Although in most cases the model proposed in this study can communicate with humans and generate acceptable results, greater distraction in the environment (such as a lot of background noise or an environment with large changes in light and shadow) may affect the results of model analysis and give unreasonable responses. Another possible reason for experiment failure lies in insufficient data training. The more complete the data training the better the model processing capabilities. Therefore, if we wish to enhance the effectiveness of the proposed model, the above two points will be very critical.

The model performance comparison proposed in this paper is mainly focused on the convergence of the model. Using different databases to train the same model will also produce completely different training results. During the interaction process between humans and the model proposed in this paper, human feelings can be very subjective, therefore human feelings are not taken into consideration in the effective analysis of the model.

This research is mainly aimed at optimizing the training efficiency of the model. In the actual application phase in the future, if it is needed to customize the model for elderly users, the first item for consideration is the establishment of a database. It is an indispensable procedure to collect and organize data on phrases and conversational nuances commonly used by elderly users. In addition, we must further study and explore the habits of using technology products by elderly users. In the future it may be possible to incorporate medical related knowledge to improve the model, thereby bridging the gap between elderly users and robots.

## 6. Conclusions

This study proposed a chatbot model that combines image and sound information and applied it to a companion chatbot. Image caption and natural language processing technologies were adopted together to design a chatbot system. The experimental results indicate that the model, combining image and sound information, is beneficial in particular chatting scenarios. Overall, the improved GRU model demonstrated superior efficacy in this application than did LSTM. The convergence in model training was also faster and more stable. In addition, the experiment verified the effectiveness and feasibility of the proposed method. In the future, this technology can be extended into the field of older adult care. It may make older people’s lives less lonely and thus improve their quality of life. The direction of this study is also helpful to aging societies.

## Figures and Tables

**Table 1:** Parameters setting of the chatbot.

Parameter	Value
Dropout Rate	0.25
GRU Cell	300
Optimizer	Adam
Loss Function	Categorical Cross Entropy

**Table 2:** Parameter settings of Image caption.

Parameter	Value
Dropout Rate	0.5
GRU Cell	500
Optimizer	Adam
Loss Function	Categorical Cross Entropy

**Figure A1:**
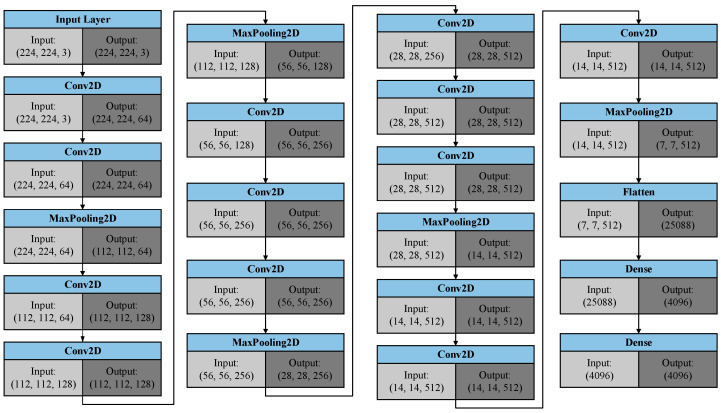
Architecture of the VGG16 model.

**Figure A2:**
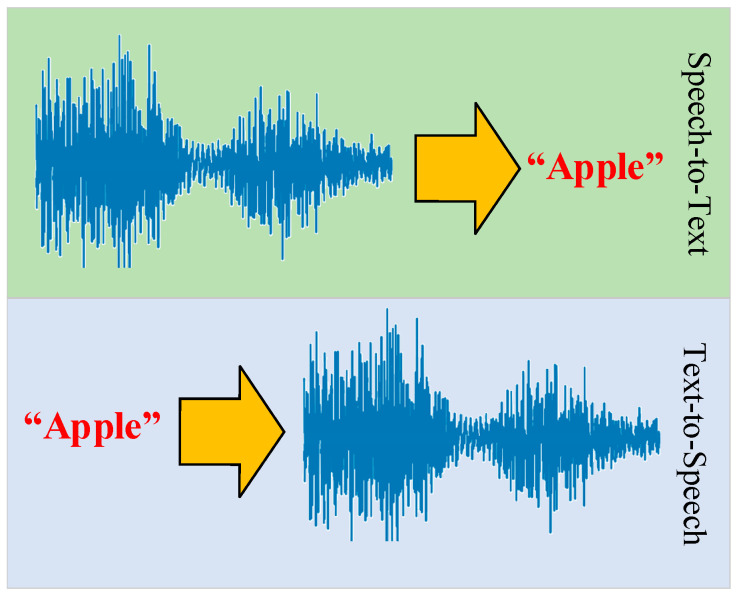
The process of Speech-to-Text and Text-to-Speech.

**Figure A3:**
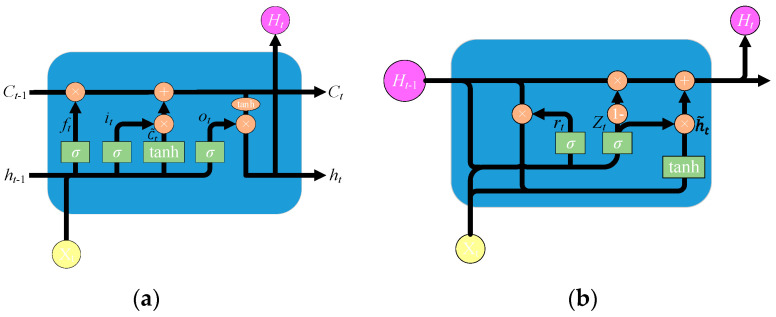
The cell structures of (**a**) LSTM; and (**b**) GRU.

## Appendix A

The VGG16 model: as presented in Figure A1, is a picture classifier with great performance and was often used when early deep neural networks first became popular.

The described methods are TTS and STT technologies, and their operational methods are presented in Figure A2. The TTS and STT technologies currently in development are already rather comprehensive. In this paper, these two technologies were used and integrated into the humanoid robot to make the robot’s responses more natural and more fluent when answering humans.

The comparison of LSTM and GRU is presented in Figure A3. The LSTM network has many determination mechanisms, or “gates”. Their function is to decide how the data must pass. For instance, the sigmoid function’s output value falls between 0 and 1; 0 means no information is allowed through, and 1 means all information is allowed. The first step in the LSTM operation is to decide what information to input. In this process, the $h_{t-1}$ and the $x_{t}$ data are read. The forget gate generates a value from 0 to 1 to decide whether the preceding data must be kept or discarded. The second step is to decide what information to update in the input gate. Next, through the tanh function calculations, we can create a new candidate value vector, ${\tilde{C}}_{t}$, and add it to the state. In the next step, updates to the new state are initiated; after $C_{t-1}$ is updated to $C_{t}$, the old state is multiplied by $f_{t}$ and the unwanted data are discarded. Then, $i_{t}*{\tilde{C}}_{t}$ is added to obtain the new candidate value. The final output value must first pass through a sigmoid function. The updated state must pass through the tanh function again, and the final outputs are multiplied to obtain the final confirmed output. The calculations are presented in (A1)–(A6).

(A1) $$f_{t}=\sigma \left(W_{f}\cdot \left[h_{t-1},x_{t}\right]+b_{f}\right)$$

(A2) $$i_{t}=\sigma \left(W_{i}\cdot \left[h_{t-1},x_{t}\right]+b_{i}\right)$$

(A3) $${\tilde{C}}_{t}=\tanh\left(W_{c}\cdot \left[h_{t-1},x_{t}\right]+b_{c}\right)$$

(A4) $$C_{t}=f_{t}*C_{t-1}+i_{t}*{\tilde{C}}_{t}$$

(A5) $$o_{t}=\sigma \left(W_{o}\cdot \left[h_{t-1},x_{t}\right]+b_{o}\right)$$

(A6) $$h_{t}=o_{t}*\tanh\left(C_{t}\right)$$

GRU is a variation on LSTM; the major difference is that it combines the forget gate and input gate into a single update gate. Other than some minor changes, the operational concepts are markedly similar. The final model is more streamlined than the original LSTM model. The calculations are presented in (A7)–(A10).

(A7) $$z_{t}=\sigma \left(W_{z}\cdot \left[h_{t-1},x_{t}\right]\right)$$

(A8) $$r_{t}=\sigma \left(W_{r}\cdot \left[h_{t-1},x_{t}\right]\right)$$

(A9) $${\tilde{h}}_{t}=\tanh\left(W\cdot \left[r_{t}*h_{t-1},x_{t}\right]\right)$$

(A10) $$h_{t}=\left(1-z_{t}\right)*h_{t-1}+z_{t}*{\tilde{h}}_{t}$$
